# Routine data analysis for moderate hemolysis interference correction in neuron specific enolase quantification

**DOI:** 10.11613/BM.2025.020802

**Published:** 2025-06-15

**Authors:** Leyre Ruiz, Tomás Munoz, Alvaro González, Estibaliz Alegre

**Affiliations:** 1 Service of Biochemistry, Clínica Universidad de Navarra, Pamplona, Spain; 2 Science Faculty, Universidad de Navarra, Pamplona, Spain; 3 Service of Biochemistry, Cancer Center Clínica Universidad de Navarra, Pamplona, Spain; 4 IdiSNA, Navarra Institute for Health Research, Pamplona, Spain

**Keywords:** NSE, hemolysis, interference, correction formula

## Abstract

**Introduction:**

Serum neuron specific enolase (NSE) is used as neuroendocrine tumor and central nervous system damage marker. It is present in variable concentrations in erythrocytes and hemolysis interferes in serum NSE quantification. Our aim was to develop a correction formula for moderate hemolysis, based on repeated patient samples instead of artificial sample doping with hemolysates.

**Materials and methods:**

We searched in laboratory informatics system for patients with sample pairs obtained within 24 h, for NSE quantification. We registered NSE and hemolytic index (NSE1 and HI1) from the first moderate hemolyzed sample (HI: 15-80), and from the second non-hemolyzed sample obtained afterwards (NSE2 and HI2). In a development cohort (N = 41), we obtained the formula NSE_calc_ = NSE1 - (0.354 x (HI1 - HI2)) - 0.162, which was later used in the validation cohort (N = 26) to calculate NSE corrected concentrations (NSE_calc_).

**Results:**

Concentrations of NSE2 differed from NSE1 (P = < 0.001) but not from NSE_calc_ (P = 0.291). In 84% samples, NSE1 had a relative bias from NSE that exceeded the 14% limit of total error allowable, with a median relative bias of 22.5%. Meanwhile, the bias between NSE2 concentrations and NSE_calc_ was - 0.4 µg/L (95% confidence interval = - 3.8 to 4.5), the relative bias was 8.3% and only 23% of samples exceeded the 14% limit. Formula usefulness was limited to moderate hemolytic samples.

**Conclusions:**

In summary, with this innovative approach, the NSE_calc_ bias is low enough to have clinical significance, so re-drawings of blood samples might be avoided. This approach also opens the possibility to correct the estimation of other magnitude concentrations affected by *in vitro* hemolysis.

## Graphical abstract

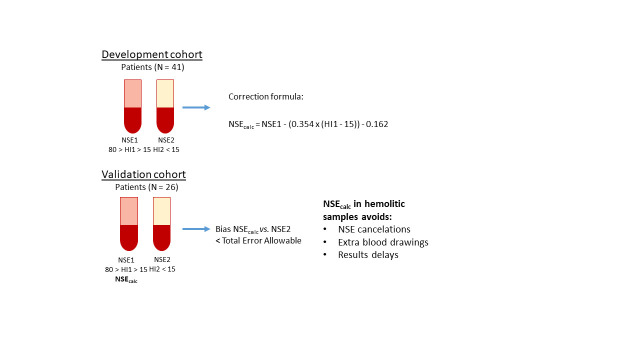

## Highlights

• A moderate hemolysis correction formula has been developed for neuron specific enolase (NSE) quantification

• Formula is based on real routine hemolyzed samples, and not in spiked-in samples

• Calculated NSE presents a minimal bias compared to non-interfered NSE

• Correction formula avoids NSE cancelations due to moderate hemolysis in the samples

• This avoids results delays and multiple blood extractions

## Introduction

Neuron specific enolase (NSE), constituted by γ-γ and α-γ dimers, is mainly present in neurons and neuroendocrine cells, but also in erythrocytes. Serum NSE is used as a marker for neuroendocrine tumors and central nervous system damage (*1*, *2*).

Serum NSE is usually quantified with automated immunoassays targeting γ isoenzyme. *In vitro* hemolysis, induced during blood drawing and processing, can interfere resulting in false NSE elevations. Consequently, assessment of hemolytic index together with NSE is highly recommended even if no hemolysis is visible (*3*).

Because of this high susceptibility for hemolysis interference, it is common practice to avoid NSE quantification in hemolyzed samples, which results in new blood drawing causing patient discomfort and results delays (*3*, *4*). Multiple studies have tried to develop formulas to correct this interference by spiking samples with increasing quantities of *in vitro*-derived hemolysates (*5*-*8*). This assumes that red blood cell NSE is constant, and may not exactly reflect naturally occurring *in vitro* hemolysis and thus, might result in inaccurate corrections.

Our aim was to develop a formula through a different approach, employing “real” hemolyzed samples obtained during clinical routine. To our knowledge, this would be the first study using this strategy.

## Materials and methods

### Subjects

We retrospectively searched in the laboratory informatics system (LIS) from Clínica Universidad de Navarra for serum samples pairs obtained from the same patient for NSE quantification (02/2019-07/2024). First sample corresponded to a moderate hemolyzed sample, with an hemolytic index (HI) = 15-80 (equivalent to 0.15-0.80 g/L of hemoglobin), whose NSE result was cancelled and not informed due to hemolysis interference. The second sample was obtained afterwards, within 24 hours after the first one, with HI < 15. We registered NSE and HI from the first hemolyzed serum (NSE1 and HI1) and from the second and not hemolyzed serum (NSE2 and HI2). No sample pair was excluded due to patient characteristics or clinical condition. A cohort of 41 sample pairs was used for formula development and another cohort of 26 sample pairs was used for formula validation.

The formula was also evaluated in an independent cohort of grossly hemolyzed samples (N = 8; HI > 80, equivalent to 0.80 g/L of hemoglobin).

No additional clinical or demographic information was recorded and the need of informed consent was waived by Ethics Committee from our institution. The study was approved by this Committee (project number 2024.130).

### Methods

Samples were drawn in serum tubes with gel (Vacutainer SST II Advance, Becton Dickinson, Franklin Lakes, USA). Samples were allowed to rest at least 30 minutes and then were centrifuged at 2460xg for 10 minutes. Each sample was analyzed independently in the same day blood was drawn.

Quantification of NSE in serum samples was performed using an electrochemiluminiscence immunoassay on the e602 module of a Cobas 8000 autoanalyzer (Roche, Basel, Switzerland). Details of the methodic of the NSE assay declared by the manufacturer include: quantification range (0.05-370 µg/L), imprecision (< 4%) and cut-off value (16.3 µg/L). In addition, according to the manufacturer, any hemolysis may interfere with the assay. Assessment of hemolysis was performed by spectrophotometry in the same autoanalyzer, providing an HI value that corresponds to mg/dL of hemoglobin, although unitless, according to the manufacturer. Interindividual variability of NSE concentration within erythrocytes was evaluated by measuring NSE in 23 hemolysates obtained as follows: a 200 µL aliquot of whole blood was separated from remanent blood tubes (5 mL BD Vacutainer K2 EDTA tubes, Becton Dickinson, Franklin Lakes, USA), once the routine analysis was completed. Erythrocytes were washed three times with 1 mL of NaCl 0.9% and then, resuspended in distilled water. Erythrocytes were then subjected to three freezing-thawing cycles to ensure hemolysis. After the final thawing process, samples were centrifuged again and diluted 1:50 in NaCl 0.9%.

Differences in NSE concentrations (∆NSE) and HI (∆HI) within each sample pair were calculated as: ∆NSE = NSE1 - NSE2 and ∆HI = HI1 - HI2.

In development cohort, Deming regression formula was obtained as: ∆NSE = A x ∆HI + B, considering ∆HI in X-axis and ∆NSE in y-axis, A the slope and B the intercept.

Subsequently, a correction formula was derived to calculate NSE concentration NSE_calc_ = NSE1 - (A x (HI1 - HI2)) - B. Afterwards, we calculated the NSE_calc_ concentration in validation cohort samples using this formula.

### Statistical analysis

We performed the statistical analysis with GraphPad Prism v10 (San Diego, USA). Due to the non-Gaussian distribution, as assessed by the D’Agostino test, NSE and HI values are presented as median and interquartile range (Q1-Q3). Spearman test was used to perform correlation analysis and Deming method for regression analysis. To compare the development and validation cohorts, HI1 and NSE1 were analyzed using the Mann-Whitney U test. Wilcoxon’s rank test was used to compare NSE1, NSE2, and NSEcalc, while bias was assessed using Bland–Altman analysis. Bias was compared with the desirable total allowable error for NSE from the European Federation of Laboratory Medicine (EFLM) database of analytical performance specification (*9*). According to this database (accessed 05/02/2025), the biological variation data are: within-subject variability (10.9%), between-subject variability (16.6%) and desirable total allowable error (14%).

## Results

### Samples included in the study

We included 67 pairs of moderate hemolytic samples with a median HI index of 25 (Q1-Q3: 18-37). These pairs were divided in between a development cohort (N = 41) and a validation cohort (N = 26). There was no difference in HI1, HI2, NSE1 or NSE2 between development and validation cohorts (Table 1). We confirmed that NSE concentration was variable in erythrocytes, by quantifying NSE in hemolyzates induced by hypotonic shock plus freezing (N = 23) where CV was 34% (data not shown).

**Table 1:** Comparison of NSE concentrations and hemolytic indexes between development and validation cohorts and in the case of validation cohort, the comparison between NSE concentration in initial hemolyzed sample (NSE1), in the second non-hemolyzed sample (NSE2) and the NSE calculated with the correction formula (NSE_calc_)

**Parameter**	**Development cohort** **(N = 41)**	**Validation cohort** **(N = 26)**	**P-value** **Development *vs*. validation**	**P-value** **In validation cohort**
HI1	23 (18-32)	24 (18-33)	0.876	
HI2	7 (4-9)	8 (6-12)	0.115	
NSE1 (µg/L)	21.2 (18.9-29.6)	22.7 (19.8-33.0)	0.550	< 0.010*
NSE2 (µg/L)	15.5 (12.9-19.7)	16.0 (14.1-24.2)	0.525	
NSEcalc (µg/L)		16.6 (15.0-24.6)		0.291**
*corresponds to NSE1 *vs*. NSE2. **corresponds to NSEcalc *vs*. NSE2. Data are expressed as median (interquartile range). P < 0.05 were considered significant. NSE - neuron specific enolase. HI - hemolytic index.				

### Hemolysis correction formula development

**Figure 1:**
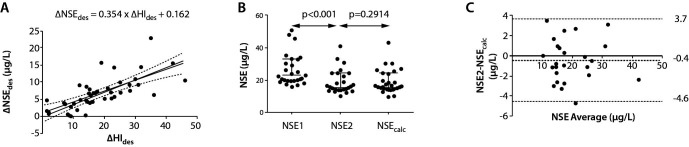
Development and validation of a correction formula for NSE quantification. **A)** Deming regression analysis in development cohort of the difference in NSE concentration (∆NSE) and the difference in hemolytic index (∆HI) among 2 consecutive blood drawings. **B)** Differences between NSE concentrations in initial hemolyzed samples (NSE1), in the non-hemolyzed samples (NSE2), and the NSE concentrations calculated with the formula applied to the initial hemolyzed samples (NSE_calc_). **C)** Bland-Altman analysis of the bias between in the non-hemolyzed samples and the calculated NSE concentrations.

In the development cohort there was a strong correlation between NSE1 and NSE2 (r = 0.75; P < 0.001) and between ∆NSE and ∆HI (r = 0.78; P < 0.001). The equation of best fitting line in the regression analysis was ∆NSE = 0.354 x ∆HI + 0.162 (Figure 1A).

Consequently, the derived hemolysis correction formula was: NSE_calc_ = NSE1 - (0.354 x (HI1 - HI2)) - 0.162

### Hemolysis correction formula validation

In validation cohort there was also a strong correlation between ∆NSE and ∆HI (r = 0.89; P < 0.001). NSE2 differed from initial NSE1 concentrations but not with NSE_calc_ derived from correction formula (Table 1; Figure 1B). Bland-Altman analysis rendered a bias of to - 0.4 µg/L (95% confidence interval (CI) = - 3.8 to 4.5) between NSE2 concentrations and NSE_calc_ (Figure 1C).

According to EFLM database of analytical performance specification, the desirable total allowable error for NSE is 14% (*9*). In 22 out of 26 samples, the relative bias of original NSE1 concentrations exceeded that limit, with a median relative bias of 22.5%. However, when considering NSE_calc_, that limit was exceeded in only 6 out of 26 samples, and the median relative bias was 8.3%.

We also evaluated the concordance between NSE2 and NSE_calc_, to classify the samples in relation to the NSE cut-off established by the manufacturer (16.3 µg/L). In validation cohort, 25 out of 26 had an initial NSE1 that exceed that cut-off while only 12 of the NSE2 concentrations exceeded it. From them, 11 samples had a NSE_calc_ above the cut-off while in one sample the calculated NSE was 15.9 µg/L. Meanwhile, NSE2 was below the cut-off in 14 samples and all of them had also a NSE_calc_ below the cut-off, except two samples with NSE concentrations very near the decision limit (18.2 and 18.6 µg/L). There was an 88.5% of agreement between classification based on NSE2 and on NSE_calc,_ with a Cohen’s kappa index of 0.769 (95% CI: 0.524 to 1.000). Once established the utility of this formula, we explored its potential utility in other conditions. Although grossly hemolyzed samples (HI > 80) are rather infrequent in routine practice, representing only 1% of our samples, we wanted to assess if this formula developed with moderate hemolyzed samples was also adequate for highly hemolyzed samples. Thus, we used it with 8 additional samples with HI ranging from 89 to 414. In this case, the NSE_calc_ presented a bias of 30.5 µg/L (95% CI: - 115 to 176), with a median relative bias of 225%. Consequently, our results show that this formula is only acceptable for HI < 80.

As the aim of this work is to avoid a second blood drawing, there would be no HI2 value to perform the correction. We considered adequate to use HI = 15 as the hemolytic value to whom refer all the corrections. For that reason, we calculated the NSE_calc15_ as the corrected NSE value for a theoretical HI = 15, and evaluated the corresponding bias. In this case, the bias was 2.8 µg/L (95% CI = - 2.1 to 7.7 µg/L) with a median relative bias of 13.1%. As previously, only 6 out of 26 samples exceeded the desirable total allowable error of 14%.

## Discussion

Quantification of NSE is interfered by minimal hemolysis, which results in that NSE results must be often annulated or postponed until a new sample is drawn. The direct effect of hemolysis in NSE concentrations has been shown by the correlation between NSE and both free hemoglobin and lactate dehydrogenase (*10*). Usually, when developing a formula to correct this hemolysis interference, the procedure is to use spiked samples (*5*-*8*). To our knowledge this is the first approach using multiple blood drawings from the same patient. We consider that this approach reflects better the hemolysis interference in NSE quantification and consequently more accurate correction formula can be derived from it.

Considering the variability in NSE content in erythrocytes, a formula based only in the effect of a limited number of hemolysates might have a limited usefulness (*8*, *11*). In the study from Nome *et al.* each serum sample was spiked with the hemolysate obtained from the same patient, which seems a more comprehensive calculation (*7*). Our study, although not using spiked samples, follows a similar approach, considering the effect of each patient hemolysis in its corresponding serum NSE measurement.

An advantage of our formula is that it exclusively relies in NSE and HI values and can be easily implementable in the LIS as a calculated NSE correction. Previous developed formulas include other magnitudes such as serum hemoglobin or NSE content in erythrocytes, which implies the requirement of an additional EDTA sample (*6*, *11*). In addition, measuring the latter is a labor-intensive process, which ultimately complicates the formula implementation in clinical routine.

The NSE_calc_ obtained with our formula did not differ from those measured in the non-hemolytic samples and the bias was much lower than that observed by other groups (*8*). Additionally, there is a high percentage of agreement between them in the classification according to the NSE cut-off, although the lower CI limit of Cohen’s kappa index shows that there is, at least, a fairly reliable agreement. We also detected that the bias between measured and calculated NSE increased in grossly hemolyzed samples, provoking that the usefulness of the formula is limited to moderate hemolyzed samples (HI < 80). This HI limit is higher than in other studies, that establish their limit at HI = 30, but lower than in the study of Liu *et al.* (up to 800) (*3*, *6*-*8*). However, we consider that that level of hemolysis is not usually reached in routine practice.

As our aim is to avoid repeated blood drawing, after formula implementation there would be no HI2 value. Thus, each lab should choose an HI2 value to which refer the NSE_calc_ concentrations. When we chose an HI of 15, the observed bias was very similar to the theoretical approach.

Previous studies recommend that the correction formula should only be applied for NSE concentrations above the cut-off (*6*, *7*). However, we consider that NSE_calc_ should be indicated whenever HI > 15, independently of NSE concentration because reliable NSE concentrations should be used during patient monitoring to detect NSE changes.

The study is based on a limited sample size, given the sample characteristics, but higher to some previous studies (*7*, *11*). However, we consider that being all of the hemolytic samples derived from the clinical routine and consequently reflecting a more physiological situation confers an additional quality and robustness to the results and conclusions derived.

In summary, we conclude that with this formula, re-drawings of blood samples due to moderate hemolysis interfering NSE quantification might be avoided. With this innovative approach the bias is low and without clinical significance. Nevertheless, the results must be informed to clinicians with a message stating that NSE results derive from a correction formula. These results also open the possibility to use the same approach to correct other molecules concentrations also affected by *in vitro* hemolysis.

## Data Availability

The data generated and analyzed in the presented study are available from the corresponding author on request.
